# Ultrasound-Guided Pericapsular Nerve Group Block for Hip Surgery: A Randomized Controlled Trial Comparing Levobupivacaine Alone Versus Levobupivacaine With Dexamethasone

**DOI:** 10.7759/cureus.84568

**Published:** 2025-05-21

**Authors:** Wasimul Hoda, Manpreet Raj, Priyanka Oraon, Neha Kumari, Alka Lakra, Chandan Hessa, Kaushal Kishore, Ladhu Lakra

**Affiliations:** 1 Anesthesiology, Rajendra Institute of Medical Sciences, Ranchi, Ranchi, IND

**Keywords:** dexamethasone, hip fracture, levobupivacaine, peng block, postoperative analgesia, randomized controlled trial, regional anesthesia

## Abstract

Background

Effective perioperative pain control in elderly patients undergoing hip fracture surgery remains a challenge due to the adverse effects of opioids and limitations of conventional nerve blocks. The ultrasound-guided pericapsular nerve group (PENG) block offers targeted analgesia with minimal motor blockade, supporting early mobilization. This study evaluates the analgesic efficacy of 0.5% levobupivacaine alone versus in combination with dexamethasone for PENG block.

Methods

In this prospective, randomized, double-blinded controlled trial, 44 adult patients scheduled for elective hip fracture surgery under spinal anesthesia were allocated into two equal groups. Group L received 20 mL of 0.5% levobupivacaine, while group LD received the same volume with 8 mg of dexamethasone. The primary outcome was the time to first rescue analgesia. Secondary outcomes included Numerical Rating Scale (NRS) scores at rest and movement, total opioid consumption in the first 48 hours postoperatively, ease of positioning for spinal anesthesia, hemodynamic stability, and anesthesiologist satisfaction.

Results

A total of 50 patients were assessed for eligibility, with 44 meeting the inclusion criteria and randomized equally into two groups (group L: 22; group LD: 22). The per-protocol analysis included 42 patients (21 in each group). Demographic characteristics were comparable between the groups. Group LD demonstrated a significantly prolonged time to first rescue analgesia (15.00 ± 2.67 hours) and second rescue analgesia (25.50 ± 3.73 hours) compared to group L (6.38 ± 1.24 hours and 12.29 ± 2.80 hours, respectively; p < 0.001). Pain scores were consistently lower at rest and during movement in the dexamethasone group at all postoperative intervals. Total opioid consumption was significantly reduced in group LD. Anesthesiologist satisfaction scores were significantly higher in group LD (p = 0.013). Side effect profiles were similar, with only one patient in Group L experiencing nausea. Both groups had comparable Ease of Spinal Positioning scores and showed no occurrence of motor blockade. Ambulation was not significantly different between the groups (p = 1.000).

Conclusion

The addition of dexamethasone to levobupivacaine in PENG block significantly enhances postoperative analgesia, facilitates patient positioning, reduces opioid requirements, and improves overall satisfaction without compromising safety. This combination may be considered a superior analgesic strategy in elderly patients undergoing hip surgery.

## Introduction

Hip fractures in the elderly represent a growing orthopedic emergency, associated with significant morbidity, mortality, and healthcare burden [1]. These injuries typically require surgical intervention to restore mobility and function [2]. However, perioperative pain management remains a critical determinant of recovery, particularly in minimizing opioid use and its associated complications.

Managing pain in this population poses unique challenges. Elderly patients are especially vulnerable to opioid-induced side effects such as nausea, sedation, and respiratory depression [3,4]. As a result, regional anesthesia has emerged as a preferred strategy, offering effective, site-specific analgesia with fewer systemic effects. Despite this, positioning for neuraxial anesthesia often causes considerable discomfort, further complicating care and impacting patient satisfaction [5].

Traditional regional techniques, such as the femoral nerve block (FNB), fascia iliaca block (FIB), and lumbar plexus block, have been widely employed in hip surgeries. However, their limitations include inconsistent coverage of the obturator nerve (ON) and accessory obturator nerve (AON), which are crucial to hip joint innervation [5-7]. Additionally, these blocks may impair motor function, delaying ambulation and increasing the risk of thromboembolism, pulmonary complications, and muscle atrophy [8,9].

Recent anatomical studies have highlighted that the anterior hip capsule, the most densely innervated portion, receives sensory input from the femoral nerve (FN), ON, and AON [10-12]. Informed by this, the pericapsular nerve group (PENG) block was introduced by Girón-Arango et al. in 2018 as an ultrasound-guided technique specifically targeting these articular branches [13,14]. The PENG block has demonstrated effective analgesia with minimal motor blockade, thus supporting early mobilization and enhancing recovery [15,16].

Compared to conventional blocks, the PENG block offers several advantages, including superior pain control, reduced opioid requirements, and a lower incidence of falls, infections, and motor weakness, all contributing to better patient outcomes and satisfaction [17]. With the global incidence of hip fractures expected to increase by 25% annually and reach 6.3 million cases by 2025, there is a growing need for effective, opioid-sparing analgesic techniques in elderly patients undergoing hip surgery [18-20]. The PENG block has shown promise in providing superior analgesia and improving patient positioning for neuraxial anesthesia. Despite its potential, no study has directly compared the use of 0.5% levobupivacaine alone versus in combination with dexamethasone for PENG block in this population.

Therefore, this randomized controlled trial was designed to evaluate the efficacy of the PENG block using 0.5% levobupivacaine alone versus in combination with dexamethasone. The primary objective was to compare the time to first rescue analgesia following block administration. Secondary objectives included comparing total opioid consumption within 48 hours postoperatively, changes in pain scores using the Numerical Rating Scale (NRS), hemodynamic stability, ease of patient positioning for spinal anesthesia, and anesthesiologist satisfaction with block performance and analgesic quality.

## Materials and methods

This study was designed as a double-blinded, randomized controlled trial aimed at comparing the efficacy and safety of two regional anesthetic techniques in patients undergoing elective hip fracture surgery under spinal anesthesia. The study was conducted over a period of 13 months at a tertiary care hospital in North India. Ethical approval for this study was obtained from the Institutional Ethics Committee of Rajendra Institute of Medical Sciences (Approval No. 112/IEC, 22-03-2024). Informed consent was obtained from all participants, or appropriately waived, in accordance with the committee's guidelines. The study was registered with the Clinical Trials Registry of India (CTRI) under the registration number CTRI/2024/05/067058. Ethical standards outlined in the Declaration of Helsinki (2013) were strictly followed.

After written informed consent, 44 patients were recruited based on the following inclusion criteria posted for hip fracture surgery under spinal anesthesia: adults aged ≥18 years, classified as American Society of Anesthesiologists (ASA) physical status I-III, and scheduled for elective hip fracture surgery under spinal anesthesia. Patients were excluded if they were classified as ASA IV or V, refused consent, had known hypersensitivity to local anesthetics, exhibited deranged coagulation profiles, had infections at the site of block placement, presented with neurological deficits (e.g., paresis, paraplegia), or had motor power <5/5 in the lower limbs.

On arrival in the operating theater, wide-bore 18G intravenous cannulas were inserted in all patients. Standard monitors were attached, and the baseline vital parameters, such as non-invasive blood pressure, heart rate (HR), and oxygen saturation (SpO₂), were recorded. Pain was assessed at rest and during movement using the NRS before spinal anesthesia as the baseline NRS score (T₀).

Randomization was performed using a computer-generated random number sequence. Allocation concealment was ensured through sequentially numbered, opaque, sealed envelopes. The study was double-blinded, and thus both the participants and investigators (including outcome assessors) were unaware of group allocations. The anesthesiologist performing the block was not involved in postoperative assessments, which were conducted by a separate blinded investigator.

Patients were randomized to one of the following groups: Group L, which received 20 mL of 0.5% levobupivacaine, and group LD, which received 20 mL of 0.5% levobupivacaine + 8 mg dexamethasone.

**Figure 1:**
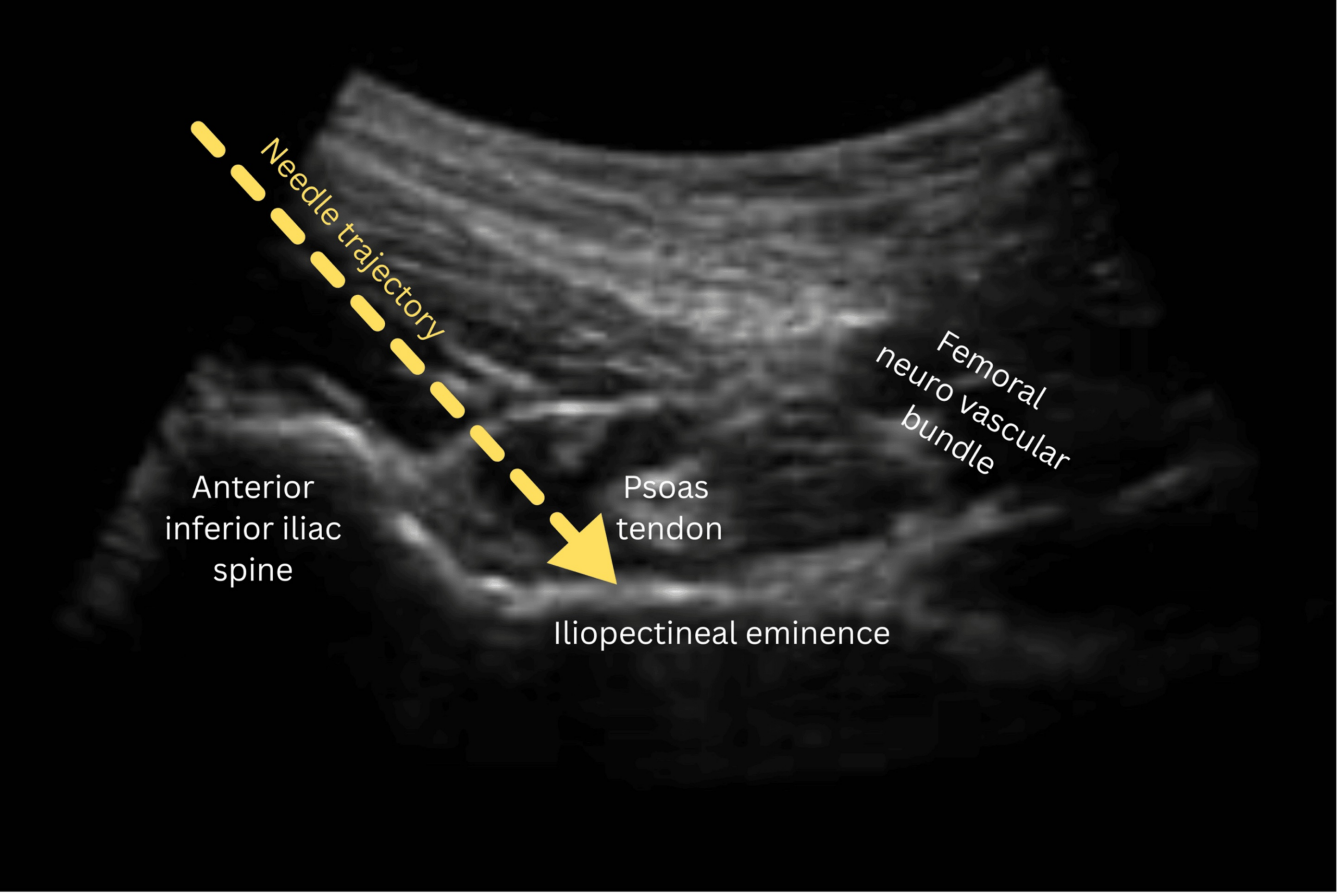
Ultrasound image showing needle trajectory for PENG block targeting the iliopsoas plane between the psoas tendon and iliopectineal eminence. PENG, pericapsular nerve group

Following standard aseptic precautions, a curvilinear transducer probe was placed at the level of the anterior superior iliac spine, parallel to the inguinal crease. The probe was gradually moved caudally until the anterior inferior iliac spine (AIIS) and psoas tendon were visualized. The target plane for local anesthetic deposition was identified between the iliopectineal eminence and the iliopsoas muscle, just inside the AIIS. A 22G spinal needle was introduced using the in-plane technique under real-time ultrasound guidance, as shown in Figure 1. After confirming negative aspiration, the 20 mL of allocated drug solution was administered incrementally. Correct spread of the injectate was confirmed by sonographic visualization and reduction in pain intensity post-injection. Pain scores were reassessed 20 minutes after block administration (TP), both at rest and during movement, prior to positioning for spinal anesthesia.

Spinal anesthesia was administered in lateral decubitus at the L3-L4 interspace using a 25G Quincke spinal needle, with 3 mL of 0.5% heavy bupivacaine injected intrathecally. The ease of spinal positioning was documented using the Ease of Spinal Positioning (EOSP) score, based on factors such as patient cooperation, spinal flexion, body mass index (BMI), and landmark visibility.

Three primary scoring tools were utilized to evaluate outcomes in this study. The NRS was used to assess pain intensity, with scores ranging from 0 (no pain) to 10 (worst imaginable pain). This scale was applied at baseline (prior to the block), 20 minutes after the PENG block (TP), and at defined postoperative intervals (6, 12, 24, and 48 hours). The EOSP score was recorded after administration of the PENG block and immediately before spinal anesthesia. This 0-3-point scale assessed four components: patient cooperation, ability to achieve spinal flexion, visibility/palpability of anatomical landmarks, and the influence of BMI, with lower scores indicating greater ease. The anesthesiologist satisfaction score was recorded immediately after spinal anesthesia positioning and before proceeding with the spinal block. This was measured on a 10-point Likert scale (1 = not satisfied, 10 = extremely satisfied) and reflected the anesthesiologist’s overall satisfaction with the ease of block performance, its analgesic efficacy, and patient comfort during positioning.

Standard non-invasive intraoperative monitoring was performed during the perioperative period. Mean arterial pressure (MAP) was monitored, and hypotension (MAP < 70 mmHg) was treated with intravenous fluids or vasopressors as needed. Intraoperative rescue analgesia included injection of tramadol 1 mg/kg IV as first-line rescue analgesia and injection of fentanyl 1 µg/kg IV as second-line rescue analgesia.

Postoperatively, pain scores were recorded at 6, 12, 24, and 48 hours, both at rest and during movement. Routine analgesia included intravenous paracetamol 1 g every 8 hours and diclofenac 75 mg every 12 hours, per institutional protocol. Additional requirements for tramadol or fentanyl were noted as part of the total rescue analgesia consumption over 48 hours.

Motor blockade, ambulation status, and potential complications (e.g., infection, neurological deficits, or allergic reactions) were monitored throughout the postoperative period. Any serious adverse events were promptly reported to the Institutional Ethics Committee and managed according to institutional guidelines.

The sample size was determined using data from a prior study by Shankar et al. [21], which reported mean times to first rescue analgesia as 1.754 ± 0.95 hours in group 1 and 2.345 ± 0.504 hours in group 2. Using a two-sided test with 80% power and a 5% significance level (Z₁-α/2 = 1.96, Z₁-β = 0.84), the pooled standard deviation (S) was calculated to be 0.7, and the mean difference (d) was 0.594. The sample size per group was derived using the following formula: n = 2 × (Z₁-α/2 + Z₁-β)² × S² / d². This yielded a minimum requirement of 20 participants per group. Considering a 10% attrition rate, the final sample size was set at 22 patients per group.

Data were entered into Microsoft Excel (Microsoft 365) and analyzed using SPSS Version 23.0 (IBM Corp., Armonk, NY). Data normality was assessed using the Shapiro-Wilk test. Continuous variables were presented as mean ± SD or median (IQR) and compared using the Student t-test or Mann-Whitney U test, depending on data distribution. Categorical data were expressed as frequencies and percentages and analyzed using the chi-square test or Fisher’s exact test as appropriate. A p-value of <0.05 was considered statistically significant.

## Results

**Figure 2:**
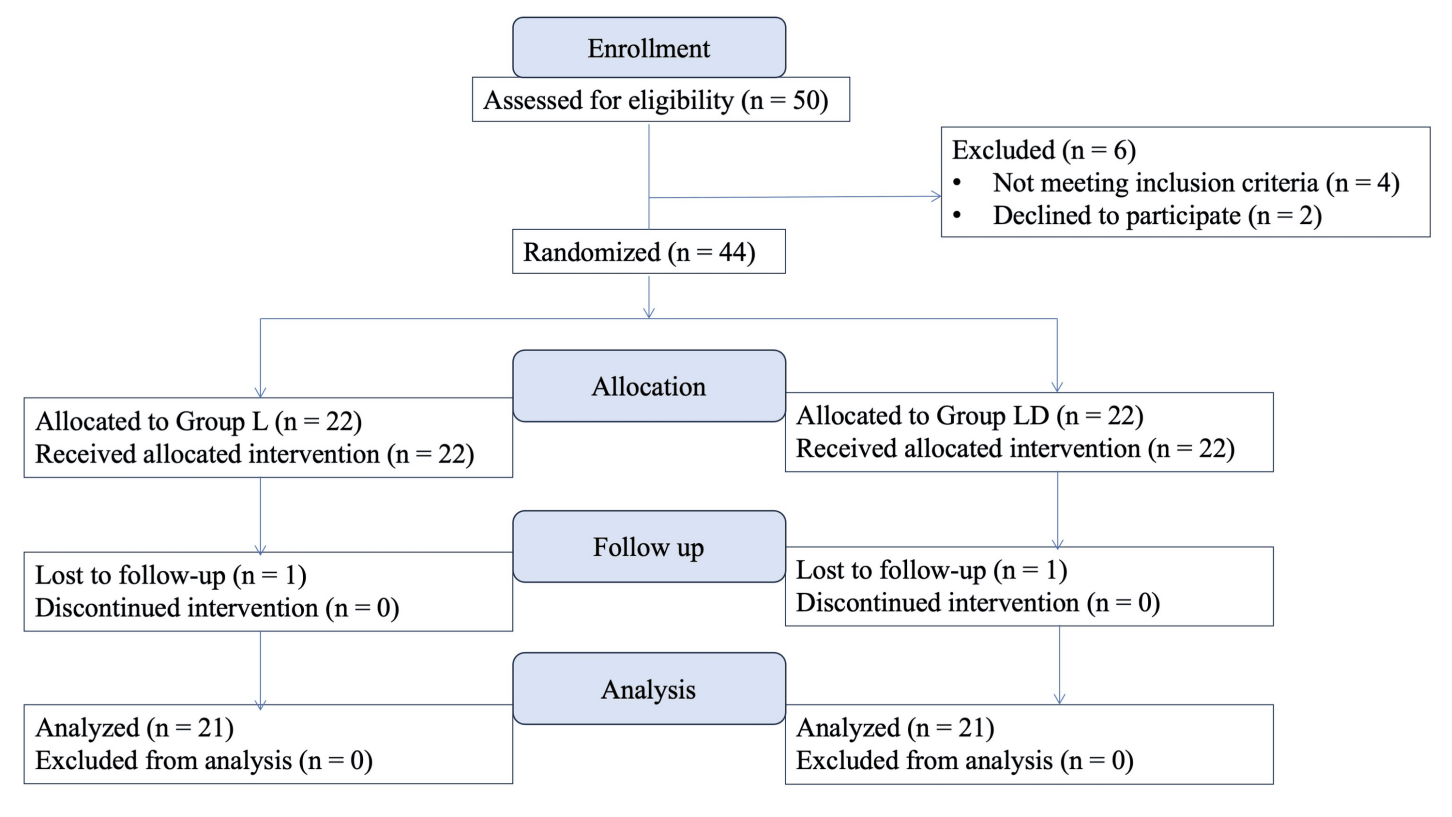
CONSORT flow diagram depicting patient enrollment, allocation, follow-up, and analysis CONSORT, Consolidated Standards of Reporting Trials; L, levobupivacaine; LD, levobupivacaine with dexamethasone

The Consolidated Standards of Reporting Trials (CONSORT) flow diagram (Figure 2) outlines the progression of participants throughout the study. Of the 50 patients assessed for eligibility, six were excluded (four due to not meeting the inclusion criteria and two due to refusal to participate). The remaining 44 patients were randomized equally into two groups: 22 in group L and 22 in group LD. All patients initially received their allocated interventions. However, one patient from each group was lost to follow-up, and the final assessment of block quality was thus completed in 21 patients per group. Consequently, a per-protocol analysis was performed on 42 patients who completed the study as per protocol.

The demographic characteristics of patients in both groups were comparable, as summarized in Table 1. The mean age was 39.71 ± 13.79 years in group L and 34.24 ± 10.70 years in group LD, with no statistically significant difference between the groups (p = 0.158). The mean body weight was also similar at 63.48 ± 7.65 kg in group L and 64.95 ± 8.06 kg in group LD (p = 0.546). The gender distribution was comparable between the two groups, with no statistically significant difference observed (p = 1.000, χ² = 0.000, df = 1, Cramér's V = 0.151). The Cramér's V of 0.151 indicates a small effect size, which is considered negligible in practical terms, confirming that gender was equally distributed across both groups.

**Table 1:** Demographic data. Values are expressed as mean ± SD. ASA, American Society of Anesthesiologists; L, levobupivacaine; LD, levobupivacaine with dexamethasone

Characteristic	Group L (n = 22)	Group LD (n = 22)	p-value
Age (years)	39.71 ± 13.79	34.24 ± 10.70	0.158
Body weight (kg)	63.48 ± 7.65	64.95 ± 8.06	0.546
Gender			
Male	17 (81.0%)	18 (85.7%)	1.000
Female	4 (19.0%)	3 (14.3%)	
ASA physical status			
ASA grade I	11 (52.4%)	17 (81.0%)	0.050
ASA grade II	10 (47.6%)	4 (19.0%)	

A borderline statistically significant difference was found in the distribution of ASA physical status between the groups, with group L having a higher proportion of ASA grade II patients n=10 (47.6%) compared to group LD n=4 (19.0%). Ideally, no significant difference should exist, as ASA was intended to be balanced between the groups. The chi-square test yielded a p-value of 0.050 (df = 1), with a Cramér’s V of 0.296, suggesting a moderate association between group allocation and ASA physical status. Despite the statistical borderline significance, the small effect size and absence of a large clinically significant difference suggest limited implications for generalizability. These findings should be interpreted with caution, as the observed difference may not have substantial practical significance.

**Figure 3:**
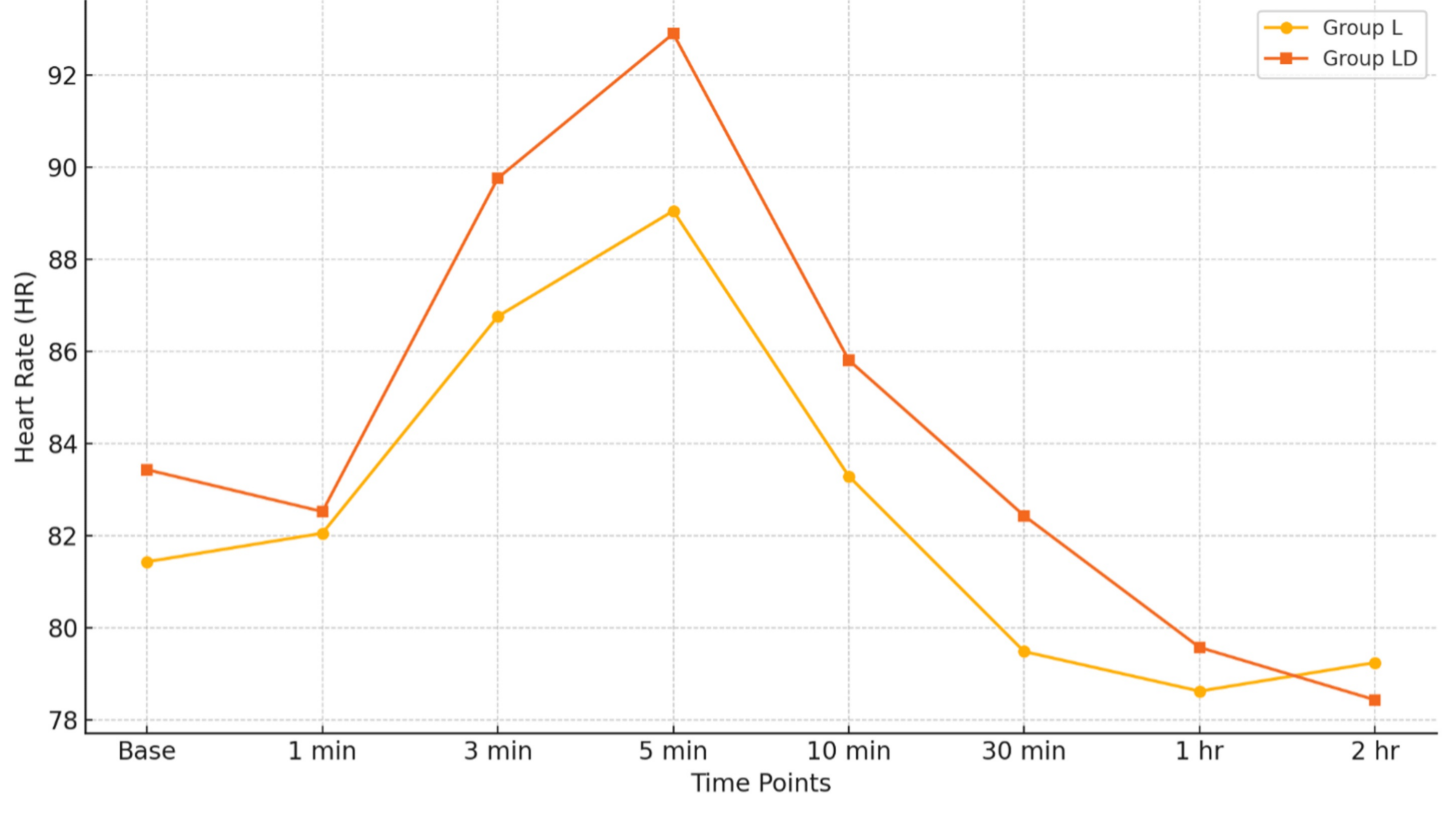
Comparison of heart rate over time between group L and group LD. L, levobupivacaine; LD, levobupivacaine with dexamethasone

**Figure 4:**
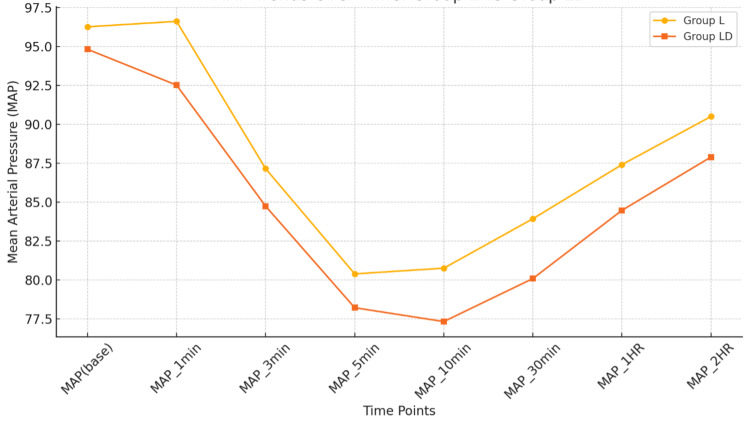
Comparison of mean arterial pressure over time between group L and group LD. L, levobupivacaine; LD, levobupivacaine with dexamethasone

Figures 3, 4 provide a graphical line plot comparison of HR and MAP at various time points in both group L and group LD, respectively. The trends in MAP and HR were found to be comparable between the two groups, with no statistically significant differences observed at any time point (p > 0.05).

**Figure 5:**
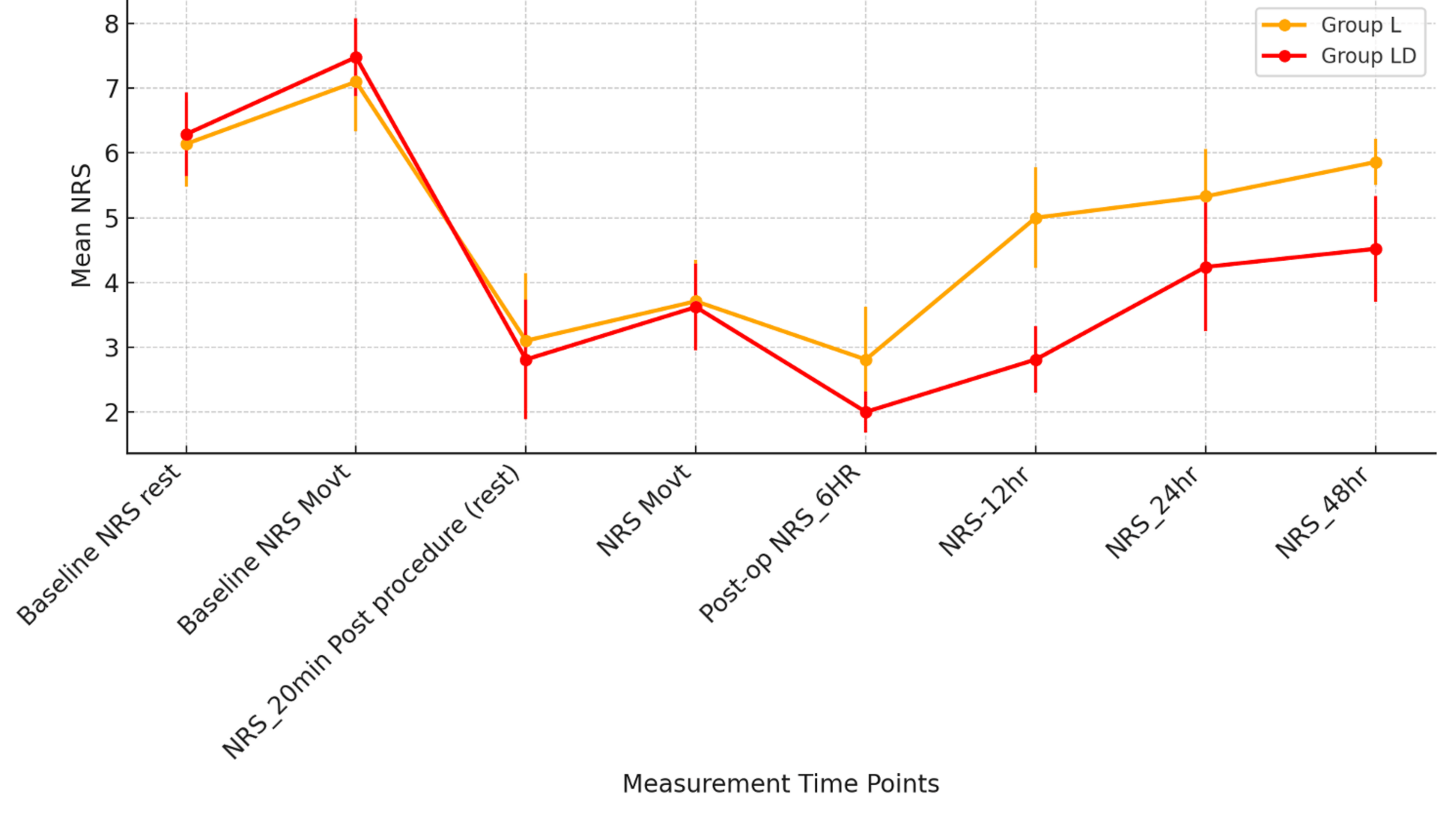
Comparison of postoperative pain scores (NRS) between group L and group LD over time. L, levobupivacaine; LD, levobupivacaine with dexamethasone; NRS, Numerical Rating Scale

Pain scores (NRS) were comparable between group L and group LD at baseline and during the immediate post-procedure period (20 minutes), both at rest and with movement (p > 0.05). However, from 6 hours postoperatively onwards, group LD demonstrated significantly lower pain scores compared to group L (p < 0.001 at 6 and 12 hours; p = 0.001 at 24 hours; p < 0.001 at 48 hours), as shown in Figure 5. These results indicate that while both groups provided effective early analgesia, group LD offered prolonged postoperative pain control.

**Figure 6:**
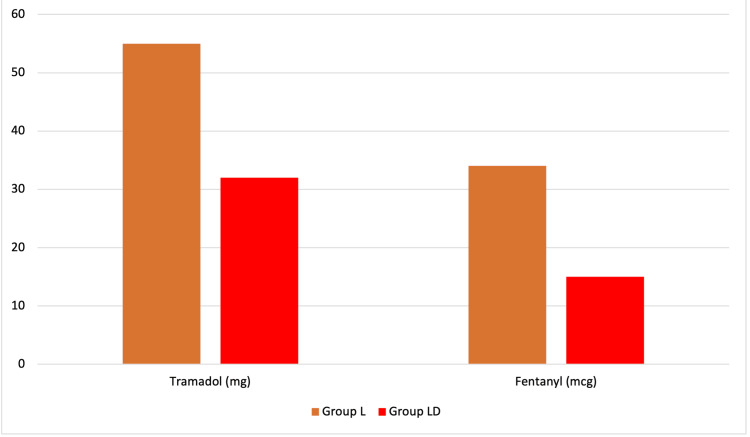
Comparison of mean opioid consumption (tramadol and fentanyl) between group L and group LD. L, levobupivacaine; LD, levobupivacaine with dexamethasone

The comparison of mean opioid consumption between the two groups showed that group L consumed significantly more opioids than group LD (Figure 6). For tramadol, the mean consumption in group L was 54.76±5.59 mg, while in group LD, it was 31.90±6.02 mg, with a statistically significant difference observed (p<0.001). Similarly, for fentanyl, group L had a mean consumption of 34.05±3.40 mcg compared to 15.00±4.26 mcg in Group LD, with a statistically significant difference as well (p<0.001) (Figure 6). These findings indicate that group L required higher doses of both tramadol and fentanyl than group LD.

**Figure 7:**
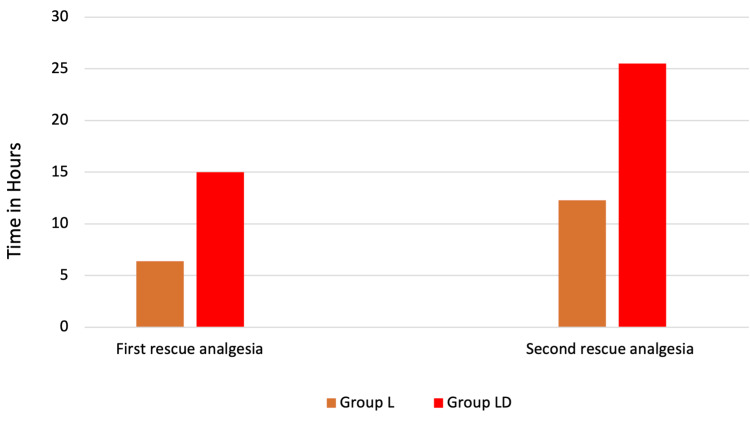
Comparison of time to first and second rescue analgesia between group L and group LD. L, levobupivacaine; LD, levobupivacaine with dexamethasone

The analysis of rescue analgesia requirements revealed statistically significant differences between the two groups. The time to first rescue analgesia was significantly earlier in group L (6.38 ± 1.24 hours) compared to group LD (15.00 ± 2.67 hours) (p < 0.001), indicating that patients in group LD experienced prolonged initial pain relief (Figure 7). Similarly, the time to second rescue analgesia was also significantly delayed in group LD (25.50 ± 3.73 hours) compared to group L (12.29 ± 2.80 hours) (p < 0.001). This further suggests that the addition of dexamethasone in group LD provided more sustained analgesia, reducing the need for additional pain medication over time (Figure 7).

Overall, these findings highlight that patients in group LD required both the first and second doses of rescue analgesia significantly later than those in group L, demonstrating the enhanced and prolonged analgesic efficacy of the intervention in group LD.

The EOSP scores were comparable between the groups with no significant difference. However, anesthesiologist satisfaction scores were significantly higher in group LD than in group L (p = 0.013), indicating greater satisfaction with the analgesic technique in group LD.

Most patients in both group L and group LD did not experience nausea or other side effects. Only one (4.8%) patient in group L reported nausea, while no patients in group LD experienced this side effect. The chi-square test revealed no significant difference between the groups in the incidence of nausea (p = 1.000, χ² = 0.000, df = 1, Cramér's V = 0.151), with a small effect size, suggesting a negligible association between group allocation and the occurrence of nausea.

Neither group L nor group LD experienced any occurrence of motor blockade, confirming that both interventions were free from this side effect. The chi-square test revealed no significant difference between the groups (χ² = 0.000, df = 1, p = 1.000, Cramér’s V = 0.0), indicating the absence of motor blockade in both groups, with no effect size to interpret. Similarly, post-operative ambulation was assessed, and no significant difference was observed between group L and group LD (χ² = 0.000, df = 1, p = 1.000, Cramér’s V = 0.0). The majority of patients in both groups were able to ambulate without difficulty, and the Cramér’s V of 0.0 further indicates that the groups had no practical difference regarding ambulation.

## Discussion

This randomized controlled trial contributes to the growing body of literature on the efficacy of PENG block in perioperative pain management for hip surgeries. Our findings demonstrate that adding dexamethasone to 0.5% levobupivacaine significantly prolongs analgesia, reduces opioid consumption, and facilitates early mobilization without causing motor impairment compared to levobupivacaine alone [22,23]. In this section, we contextualize these results within existing literature, explore potential mechanisms, and discuss the clinical implications, as well as the study's strengths, limitations, and future research directions.

The primary outcome, time to first rescue analgesia, was significantly longer in the group receiving levobupivacaine with dexamethasone compared to levobupivacaine alone. Patients in the combination group also experienced consistently lower pain scores during positioning and throughout the postoperative period, consumed fewer opioids, and had better anesthesiologist satisfaction. These results suggest that dexamethasone, when added to levobupivacaine, enhances the efficacy of the PENG block, providing superior pain relief and improved patient outcomes [22,23].

Our findings are consistent with previous studies highlighting the effectiveness of the PENG block in hip fracture surgeries. Girón-Arango et al. first demonstrated its ability to selectively anesthetize the FN, ON, and AON, providing effective anterior hip analgesia with minimal motor blockade [15,18]. Swetha et al. and Yadav et al. further supported the PENG block as a safe, feasible alternative to traditional blocks, promoting better outcomes and reduced opioid intake [24,25]. Salgado-García et al. reported that both levobupivacaine- and ropivacaine-based PENG blocks offer effective analgesia with minimal opioid use in elderly hip fracture patients [26]. Our study builds on these findings by incorporating dexamethasone as an adjuvant, a strategy that has been shown to enhance the duration of local anesthetic effects.

The enhanced analgesia observed with dexamethasone is likely multifactorial. As a corticosteroid with potent anti-inflammatory properties, dexamethasone may act through several mechanisms: inhibition of prostaglandin synthesis, suppression of ectopic neuronal discharge, and vasoconstriction, which delays the systemic absorption of local anesthetics. These effects collectively contribute to prolonged sensory blockade and reduced postoperative hyperalgesia. Pascarella et al. have demonstrated that dexamethasone improves both the duration and quality of analgesia in regional blocks, findings consistent with our results [23]. However, further research is needed to evaluate the long-term safety of perineural dexamethasone, particularly when used with levobupivacaine [27].

Clinically, incorporating dexamethasone into the PENG block offers several advantages. Prolonged analgesia reduces the need for systemic opioids, thereby minimizing opioid-related side effects such as nausea, sedation, constipation, and respiratory depression - issues particularly pertinent in elderly patients. Moreover, the motor-sparing nature of the PENG block facilitates early postoperative mobilization, which is essential in preventing complications such as thromboembolism and muscle deconditioning [27-29]. These benefits align with the Enhanced Recovery After Surgery (ERAS) protocols, which emphasize reducing opioid use and promoting early mobility [27,29]. Given the increasing prevalence of hip fractures, especially in older adults, these findings highlight the potential for improving perioperative care and reducing healthcare burdens.

Compared to traditional techniques such as FIB and FNB, the PENG block provides superior analgesia for hip joint pain while preserving quadriceps strength. FIB and FNB often fail to adequately block the ON, resulting in incomplete analgesia and delayed rehabilitation [29]. In contrast, the PENG block targets the FN, ON, and AON, providing more comprehensive anterior hip analgesia [18]. The findings of this study further support the PENG block as a preferred technique for hip surgery, underscoring its role in multimodal analgesia regimens [24,25].

The strengths of this study include its randomized controlled design and focus on a relatively novel regional anesthesia technique. The use of objective, validated outcome measures enhances the reliability of our findings. However, there are some limitations. The study was conducted at a single center with a modest sample size, which may limit the generalizability of the results. Higher ASA grade patients were excluded, restricting applicability to more medically complex populations. Surgical duration was not recorded. However, most surgeries were performed by the same experienced surgeon using a consistent technique. Therefore, surgical times were likely similar across both groups. Nonetheless, the lack of precise data limits the evaluation of its impact on analgesic outcomes. The study focused on short-term postoperative results, leaving long-term recovery and functional outcomes unexplored. Although no adverse effects related to dexamethasone were observed, the study was not powered to detect rare or delayed complications from perineural corticosteroid use.

Future research should include multicenter trials with larger, more diverse patient populations, incorporating higher ASA grades to enhance external validity. Long-term follow-up assessing functional recovery, quality of life, and safety outcomes after perineural dexamethasone use is also warranted. Comparative studies evaluating the PENG block against other regional anesthesia techniques across various surgical settings will further clarify its clinical value.

## Conclusions

Our study highlights the ultrasound-guided PENG block as an effective regional anesthesia technique for hip surgeries. The addition of dexamethasone to levobupivacaine enhances analgesic duration, reduces opioid consumption, and promotes early mobilization - key components of effective perioperative care. Given its favorable risk-benefit profile and alignment with ERAS protocols, the PENG block should be considered for routine use in hip surgery, particularly in elderly patients. Future large-scale, multicenter trials are necessary to further establish its role in contemporary anesthesia practice.
